# Ambulatory Surgery Protocol for Endoscopic Endonasal Resection of Pituitary Adenomas: A Prospective Single-arm Trial with Initial Implementation Experience

**DOI:** 10.1038/s41598-020-66826-9

**Published:** 2020-06-16

**Authors:** Yang Liu, Tao Zheng, Wenhai Lv, Long Chen, Binfang Zhao, Xue Jiang, Lin Ye, Liang Qu, Lanfu Zhao, Yufu Zhang, Yafei Xue, Lei Chen, Bolin Liu, Yingxi Wu, Zhengmin Li, Jiangtao Niu, Ruigang Li, Yan Qu, Guodong Gao, Yuan Wang, Shiming He

**Affiliations:** 1 Department of Neurosurgery, Tangdu Hospital, Fourth Military Medical University, Xi’an, China; 2 Department of Neurology, No.988 Hospital of Joint Logistic Support Force, Zheng Zhou, China; 3 Department of Nutrition, Tangdu Hospital, Fourth Military Medical University, Xi’an, China; 4 Department of Neurosurgery, Xi’an International Medical Center, Xi’an, China; 5 Department of Anesthesiology, Tangdu Hospital, Fourth Military Medical University, Xi’an, China

**Keywords:** Clinical trials, CNS cancer

## Abstract

Endoscopic endonasal transsphenoidal resection has been accepted as a routine therapy for pituitary adenoma, but the postoperative hospital stay is typically several days long. With the advantages of reduced cost and improved patient satisfaction, the application of ambulatory surgery (AS) has developed rapidly. However, AS was still rarely adopted in neurosurgery. Here we designed an AS treatment protocol for pituitary adenoma with the endoscopic endonasal approach (EEA), and reported our initial experiences regarding the safety and efficacy of the AS protocol. 63 patients who presented with pituitary adenoma were screened at the Department of Neurosurgery, Tangdu Hospital from July to September, 2017. A total of 20 pituitary adenoma patients who met the inclusion criteria underwent EEA surgery using this evidence-based AS protocol, which emphasized adequate assessment for eligibility, full preparation to minimize invasiveness, enhanced recovery, and active perioperative patient education. Of the 20 patients enrolled, 18 were discharged on the afternoon of the operation day with a median total length of stay (LOS) of 31 hours (range, 29–32) hours. The median LOS after surgery was 6.5 (range, 5–8) hours. Two patients were transferred from the AS protocol to conventional care due to intraoperative cerebrospinal fluid leakage (one case) and an unsatisfying post-anesthetic discharge score (one case). Complications included transient and reversible mild postoperative nausea and vomiting [visual analog scale (VAS) score <3], headache (VAS score <3) after the operation or early after discharge. No patient was readmitted. Our results supported the safety and efficacy of the AS protocol for pituitary adenoma patients undergoing EEA resection among eligible patients, and further evaluation of this protocol in controlled studies with a larger sample size is warranted.

## Introduction

Current advances in perioperative surgical concepts and technologies promote the advent and prevalence of ambulatory surgery (AS) in several surgical fields. The use of AS has accounted for >60% of all surgeries performed in the United States, with improved patient satisfaction and reduced costs compared to traditional surgery^1^. In the field of neurosurgery, although attempts had been made to resect supratentorial brain tumors and clip unruptured cerebral aneurysms on an outpatient basis^2,3^. AS remains not widely adopted.

The endoscopic endonasal approach (EEA) for the resection of pituitary adenomas has gained increasing acceptance over the past few decades. Evidence suggested that EEA features shorter operative time, minor surgical hemorrhage, less post-operative pain, higher quality of life, and a shorter length of stay (LOS) with a lower incidence of postoperative complications^4^. These characteristics are consistent with the basic principles of AS^5^.

In our institution, as a large tertiary care hospital and one of the largest neurosurgery centers in China, we have implemented an evidence-based enhanced recovery after surgery protocol (ERAS) for elective craniotomy, which has been observed to lead to a shorter LOS and lower morbidity rates^6^. Building upon our neurosurgical ERAS protocol and experience, after reviewing the current literature on successful AS protocols in other surgical fields, we initiated an ambulatory protocol for pitutary adenomas resection via the EEA for selected patients. Here we reported and analyzed our initial experiences regarding the safety and efficacy of the AS protocol.

## Participants and Methods

### Participants recruitment

This prospective single-arm single-center trial examined whether pituitary adenoma patients can be treated safely and effectively by AS protocol. The trial was registered with the Chinese Clinical Trial Registry (ChiCTR-ONC-17012019) with the date of registration as 17/07/2017. From July to September 2017, patients aged 18–65 years who presented with pituitary adenoma at the Department of Neurosurgery, Tangdu Hospital, were assessed for eligibility for the AS protocol. Considering the patients’ complicated conditions, only those with nonfunctioning adenomas <3 cm in diameter (Knosp grade 0–2, Hardy Wilson grade I, IIA, or IIB) and with acceptable status [American Society of Anesthesiologists (ASA) physical status grade 1 or 2] were considered eligible and recruited into the study. Correspondingly, patients with functional adenomas or large nonfunctional adenomas (>3 cm in diameter) and moderate to severe comorbidities that could impact the postoperative recovery were excluded. In addition, patients with local infection near the surgical field and those lacking proper caregivers were also considered unsuitable for AS protocol.

Patients consented to conversion to the conventional neurosurgical inpatient treatment protocol at any time based on their own will and their general status during and after the operation. Patients who presented with complications such as vision decline, diabetes insipidus (DI), inability to feed, or moderate to severe headache [visual analog scale(VAS) score >4] were also considered unsuitable for same-day discharge. Meanwhile, patients were transferred to conventional inpatient treatment protocol and considered dropped out from the AS protocol when any of the following occurred: surgical duration >3 hours, intraoperative blood loss >500 mL, and sellar perforation or cerebrospinal fluid (CSF) leak.

Although certain modifications were made to adapt the situation of endonasal transsphenoidal surgery, the post-anesthetic discharge scoring system (PADSS) was introduced to stratify patients postoperatively. In our protocol, computed tomography (CT) was used to assess the extent of the hemorrhage. When no high-intensity bleeding was observed in the operative area or nasal cavity, it was scored 2. When a small amount of bleeding was observed only in the nasal cavity, it was scored 1. When a large amount of bleeding was observed in the nasal cavity, it was scored 0. Meanwhile, patients were regarded unsuitable for discharge and required admission for further monitoring.

Only patients who showed an acceptable general status (PADSS ≥9) with no intra- or postoperative complications mentioned above were allowed to be discharged on the operation day or early the next morning. Patients who did not meet the discharge criteria were admitted for routine inpatient management.

### Compliance with ethical standards

Informed consent was obtained from all individual participants or their legal representatives. The analysis and usage of patient information for this study were approved by the Ethical Committee of Tangdu Hospital. The methods were performed in accordance with approved guidelines.

### Preoperative preparations

According to the AS protocol for pituitary adenoma, patients and their family members were informed about the expectation for discharge on the operation day. The feasibility to discharge with a mild headache or other minor complications was also clarified during further education. The patients and their caregivers were educated in detail regarding the perioperative care program, including the demonstration of nasal irrigation with saline and signs and symptoms of potential complications, including fever, DI, CSF leak, meningitis, and visual decline. They also agreed to participate in daily telephone follow-up for at least one week postoperatively. A clinical outpatient revisit was required 4 weeks after surgery.

The patients were instructed to abstain from alcohol and cigarettes one week before the operation and encouraged to perform physical exercise to improve their pulmonary function. Blood pressure and serum glucose were controlled in the target range, and diet was guided to improve nutrition status.

Patients were admitted the day before the operation in accordance with our hospital’s current policy. Since the patients’ endocrine hormones had been assessed as part of a pre-enrollment evaluation, we did not perform this assessment upon admission. Prolonged fasting from midnight was not obligated and oral carbohydrate loading (i.e., maltodextrin fructose solution, 400 mL) was applied at least 2 hours prior to the surgery. Prophylactic antibiotics were administered one hour prior to the operation. Atropine and dexamethasone were used to reduce gland secretion and minimize the stress response. Serotonin receptor antagonists were routinely administered to prevent postoperative nausea and vomiting (PONV). Preoperative anxiolytics and long-acting sedatives were not used to avoid postoperative sedation.

### Anesthetic and surgical management

The operation was performed at 8:00 am. Optimized perioperative anesthesia was crucial to decreasing the surgery duration and incidence of complications. In our protocol, the combination of general anesthetics with a short half-life and long-lasting local anesthetics was applied. The fluid intake was minimized with careful monitoring of blood pressure and intravascular fluid volume, and a core temperature of 36–37°C was maintained using warmed solutions and insulation blankets to prevent hypothermia. Indwelling urinary catheters were placed in all patients to monitor urine output.

The endoscopic endonasal resection of the pituitary adenoma was performed using a routine 2-dimensional endoscopic system. A mucosal flap with vascular pedicle and multi-layer reconstruction protocols were applied to reduce the incidence of a CSF leak. It was worth noting that surgical manipulation was careful and hemostasis was achieved. Lumbar drainage was not included in the AS protocol since it can increase the LOS. Postoperatively, the patient was monitored in the recovery room until fully awake and hemodynamically stable with a Steward Scoring System score >4. Thereafter, a thorough neurological examination was performed to detect any surgery-related dysfunction. Patients with an intact neurological status were transferred to an AS unit. The urinary catheter was removed soon after the operation to prevent delayed ambulation.

Postoperatively, the opium analgesic was not routinely prescribed unless a patient’s pain VAS score was >7, and non-opioid analgesia strategies were applied according to the patient’s VAS score in our center.

When a surgical duration was more than 3 hours, or CSF leak, vision decline, intraoperative hemorrhage >500 mL, or other neurological complications occurred, the patients were converted to traditional neurosurgical inpatient treatment management. If postoperative pain was well-controlled with oral medications (VAS score <4) without hemorrhageor a CSF leak, patients were encouraged to intake a liquid and pureed diet orally early after regaining consciousness. A postoperative CT scan was performed three hours after surgery for each patient. Early ambulation was strictly monitored and achieved with the help of medical staff whenever necessary. After the operation, patients underwent comprehensive evaluations, including the conditions of hemodynamic stability, neurological status, and the daily self-care ability assessed by Karnofsky Performance Scale (KPS) and PADSS. When KPS score ≥70 and PADSS score ≥9, patients were considered eligible to be discharged home following the AS protocol. The manifestations of potential complications and ways to recognize them were re-emphasized to patients and their caregivers before discharging.

### Follow-up

Routinely all patients were recommended to stay with their caregiver(s) in areas no more than 2 hours away from the hospital for the first three nights after the surgery. Telephone follow-up was conducted every day for the first week after discharging to monitor the incidence of possible complications^7,8^. Common complications were cerebrospinal fluid fistula, diabetes insipidus, and meningitis^9,10^, and they were of special concern. The follow-up was managed by our AS team in which at least one neurosurgeon and skilled inpatient nurse were contained. Temperature, fluid intake and output, urine color were concerned for manifestations of possible fluid and electrolyte disturbance. Symptoms of hyponatremia were of special concern. These symptoms include lethargy, altered cognitive or mental status, recurrent or aggravated headache and nausea/vomiting days after the operation. Whenever manifestations such as polyuria, polydipsia, or pale-colored urine occurred, or hyponatremia symptoms were suspected, patients were recommended to go to the outpatient green channel for further evaluation as soon as possible^11^. By postoperative day 8, patients received thorough assessment at the neurosurgery clinic. During this meet, general physical evaluation, a nasal endoscopic examination, blood biochemical test and urine assessment were performed. Pituitary magnetic resonance imaging (MRI), visual tests, and endocrine analyses including hypophysial hormone, thyroid function, gonadal hormone, and cortisol were performed by one month after discharge to evaluate pituitary function and the degree of the operation incision. Pituitary MRI was recommended 3–6 months after discharging for further effectiveness and safety evaluation of the operation.

During follow-up period, a 24-hour telephone hotline and outpatient green channel was always available whenever any complications were suspected. For relatively severe complications such as CSF rhinorrhea, emergency service was provided, including immediate admission for full evaluation and further adequate management.

## Results

### Patient characteristics

Until September 2017, a total of 63 patients aged from 18 to 65 years were screened for eligibility for our AS protocol. Their clinical description are listed in Table 1. The median age was 47 years old, and the mean maximum diameter was 2.7 ± 0.8 cm (ranged 1.2–4.9 cm). Among them, 36 cases were female (57%). Most of their adenomas were nonfunctioning or prolactin secreting adenomas, accounting for 49% (31 cases) and 38% (24 cases) of the cohort respectively. Additionally, there were six growth hormone secreting adenoma cases (10%), and two adrenocorticotropic hormone secreting adenomas cases (3%).

**Table 1:** Clinical data of 63 patients screened for pituitary adenoma resection.

Parameters	Values
Median age in years (range)	47 (18–65)
Female sex, no. (%)	36(57%)
Median BMI (range, kg/m^2^)	23.7 (17.0–28.9)
Mean maximum diameter(range, cm)	2.7 ± 0.8 (1.2–4.9)
Knosp grade	
Knosp grade 1, no. (%)	12(19%)
Knosp grade 2, no. (%)	29(46%)
Knosp grade 3, no. (%)	14 (22%)
Knosp grade 4, no. (%)	8(13%)
Nonfunctioning	31(49%)
Endoscopy surgery in AS protocol	20
Endoscopy surgery in non-AS protocol	11
Prolactin secreting	24(38%)
Dopamine agonist	13
Endoscopy surgery in non-AS protocol	11
Growth hormone secreting	6(10%)
Endoscopy surgery in non-AS protocol	6
Adrenocorticotropic hormone secreting	2(3%)
Endoscopy surgery in non-AS protocol	2

Among the 63 cases, 20 patients met the inclusive criteria and were enrolled in this AS protocol, including seven male (35%) and thirteen female (65%). Their clinical data are shown in Table 2. The median age was 38 years old (range, 22–62 years old), and the median body mass index (BMI) was 21.8 kg/m^2^ (range, 17.1–27.9 kg/m^2^). On the clinical manifestation, eleven patients presented with visual field defects, seven cases showed disturbing headaches which cannot be interpreted with other etiology (two fell into both categories), and two cases presented with menstrual dysfunction. It was worth noting that another two patients showed no evident clinical symptom but they were enrolled because of the propensity for adenoma enlargement. On the characters of preoperative high-resolution MRI imaging, their mean adenoma diameter was 2.2 ± 0.3 (range, 1.6–2.7) cm, and five (25%) cases were categorized into Knosp grade 1 and fifteen (75%) were categorized into Knosp grade 2. In our cohort, no patient was Hardy-Wilson grade I, while four cases (20%) were Grade IIa and sixteen cases (80%) were Grade IIb. On the preoperative concomitant status evaluation, fifteen patients showed no obvious comorbidity and the other five patients (25%) had mild systemic disease, such as chronic obstructive pulmonary disease, mild coronary artery disease, well-controlled diabetes, or hypertension requiring medication. In addition, a total of five patients were current smokers.

**Table 2:** Demographic of 20 patients receiving ambulatory surgery with endoscopic endonasal approach for pituitary adenoma resection.

Parameters	Values
Median age in years (range)	38 (22–62)
Female sex, no. (%)	13(65%)
Median BMI (range, kg/m^2^)	21.8 (17.1–27.9)
Clinical presentations	
Field defects	11 (55%)
Headache	7 (35%)
Menstrual dysfunction	2(10%)
No evident symptom	2 (10%)
Mean maximum diameter(range, cm)	2.2 ± 0.3 (1.6–2.7)
Knosp grade	
Knosp grade 1, no. (%)	5(25%)
Knosp grade 2, no. (%)	15(75%)
Hardy-Wilson grade	
Grade I, no. (%)	0(0%)
Grade II, no. (%)	20(100%)
Grade IIa, no. (%)	4(20%)
Grade IIb, no. (%)	16(80%)
ASA class	
I, no. (%)	15 (75%)
II, no. (%)	5 (25%)
Preoperative health and comorbidities	
Current smoker, no. (%)	5(25%)
Diabetes, no. (%)	2(10%)
Obstructive sleep apnea syndrome (Apnea–hypopnea index <15), no. (%)	1 (5%)
Hypertension requiring medication, no. (%)	3(15%)
Coronary artery disease, no. (%)	1 (5%)

*ASA grade: American Society of Anesthesiologists physical status grade.

Among the 43 patients who did not meet the inclusive criteria, 30 patients received EEA operation and were managed with traditional non-AS protocol. The other thirteen patients who carried prolactin secreting adenomas were prescribed with dopamine agonist and were recommended for outpatient follow-up regularly.

### Surgical resection and hospital LOS

Adenomas were completely resected in all patients via endoscopic endonasal approach. By the end of the sixth month after discharging, operation sites in a total of seventeen patients were re-imaged by pituitary MRI, and no relapse of the adenomas was observed. This further supported the effectiveness of AS procedure in dealing with adenoma in selected patients.

On the operation data, the median surgical duration was 82 min (range, 70–115 min); one operation (5%) lasted longer than 100 min because of the occurrence of CSF rhinorrhea. After careful and thorough sellar reconstruction, the AS protocol was discontinued and the patient was admitted to the neurosurgery ward for further treatment. The patient was discharged with a LOS of three days, and no fever or CSF rhinorrhea was reported during a one-month follow-up.

On the early post-operative outcomes, a total of eighteen patients (90%) were discharged on the operation day as expected. Their total LOS was a median 31 hours (range, 29–32 hours), while their LOS after surgery was a median 6.5 hours (range, 5–8 hours). Among them, 12 patients were discharged from the hospital with a PADSS score of 9 for mild headache, while eight patients left with a PADSS score of 10. Most patients could lead normal activity with effort (KPS = 80; 12 cases). The discharging KPS score was 90 in five cases and 70 in three cases. In addition to the cases with intraoperative CSF leaks, one case (5%) was dropped from the AS protocol and transferred to conventional neurosurgical inpatient care due to moderate PONV with a poorly controlled headache. He was discharged with a total LOS of two days once his general condition met the discharge criteria. LOS and perioperative outcomes were summarized in Table 3.

**Table 3:** Length of stay and perioperative outcomes.

Parameters	Values
Median duration of the operation in min (min, 1stQ, 3rd Q, max)	82 (70,75, 85,115)
Patients Transferred to inpatient care (no.)	2
CSF rhinorrhea during the operation	1
Severe PONV after operation	0
Poorly controlled headache (VAS scoreå 7)	1
Patients discharged on the operation day (no.)	18
Median LOS after surgery in hrs (min, 1stQ, 3rd Q, max)	6.5 (5, 6, 7.5, 8)
Median total LOS in hrs (min, 1stQ, 3rd Q, max)	31 (29, 31, 31, 32)
Discharge PADSS score	
PADSS = 9 (no.)	12
PADSS = 10 (no.)	8
Discharge KPS score	
KPS = 70	3
KPS = 80	12
KPS = 90	5
KPS = 100	0
Postoperative complications within 8 hours after surgery	
Mild PONV (VAS <3)	4
Minor bleeding	0
Mild headache (VAS <3)	6
Visual impairment	0
Transient diabetes insipidus	0
Follow-up postoperative complications (1 month)	
Mild PONV (VAS <3)	0
Minor bleeding	0
Mild headache (VAS <3)	2
Visual impairment	0
Transient diabetes insipidus	0
Olfactory impairment	3
Re-admission	0

*LOS: Length of stay.

PADSS: Post-anesthetic discharge scoring system.

KPS: Karnofsky Performance Score.

PONV: Postoperative nausea and vomiting.

VAS: Visual analog scale.

CSF: Cerebrospinal fluid.

Although some complications including mild headache and nasal ventilation were acceptable when patients were discharged, these symptoms lasted momentarily for a period of 1–2 days. No patients showed postoperative symptoms of DI or nasal bleeding. This was confirmed in clinical visit one month later.

At the point of one month postoperative follow-up, no complications were observed in any patient, including CSF rhinorrhea, endocrine dysfunction, local infection, and visual. However, olfactory impairment was observed in 3 cases. No patient was re-admitted for complications.

A total of 30 patients received EEA operation and were managed by traditional non-AS protocol. The LOS and postoperative outcomes of these 30 patients were briefly described in Supplementary Material.

## Discussion

Shorter hospital stays could benefit patients with a lower cost and better medical service experience^12,13^. Therefore, decreasing the hospital LOS in the precondition that both postoperative complications and readmission remained favorable is beneficial for health care providers, the medical insurance system, and patients. It was reported that the mean LOS for primarily diagnosed pituitary adenoma patients was 4.8 days in 2011 in the United States^14^. J.G. Thomas *et al*. further reduced the average LOS to 1.16 ± 0.55 days in a prospective trial in a cohort of 50 consecutive patients who were managed with a short-stay perioperative care protocol^15^. In his report, though postoperative diabetes insipidus occurred in 16% of patients, patients were discharge on schedule and readmission was required in two patients for delayed presentation of a CSF leak.

Ambulatory surgery, or day surgery, is often associated with higher patient satisfaction and less exposure to nosocomial comorbidities. It has been increasingly accepted and generalized in several surgical fields and many countries worldwide in recent years^16–18^. In terms of effectiveness and safety, low overall mortality and re-admission rates were reported within 2–3 days after surgery and in a follow-up period of 30–60 days^19–22^. In the field of neurosurgery, although the feasibility of AS was also reported in relatively small cohorts of patients who underwent a craniotomy for brain tumors and even in children who underwent ventriculoperitoneal shunt for hydrocephalus and spinal bifida repair^16,23–25^, it remained challenging to balance the significantly reduced LOS and patient safety/outcome. After reviewing the current rare application of AS in neurosurgery, we noticed several factors influencing AS progress, such as the unique sophisticated neurosurgical manipulations, procedural complexity, and the concerns and strategies for managing potential surgery-related complications.

Here we described an AS protocol for selective patients with pituitary adenomas. In our cohort treated with this current protocol, a total of 18 patients (90%) were discharged within 24 hours; their LOS after surgery was a median 6.5 (range, 5–8) hours. Our study initially demonstrated the feasibility of pituitary adenoma resection with the EEA following this AS procedure. In our protocol, the established endoscopic surgical technique and sound patient communication and education during perioperative period with the ERAS concept were considered the preliminary factors for its successful implementation.

For an experienced surgeon, endoscopic procedures improve surgical instrument maneuverability owing to an advanced integrated optical system and surgical approach flexibility^4^. For patients, endoscopic surgery is minimally invasive to the cranial bones and soft tissues, which helps reduce postoperative discomfort and analgesic use^4^. Evidence indicates that endoscopic pituitary surgery could bring quick patient recovery while remaining effective and safe like the traditional transcranial approach^26,27^. To adequately reduce LOS, close and effective cooperation among clinicians, anesthesiologists, inpatient and operative nurses, and nutrition services is critical^10^. Based on our experience, practiced surgical manipulation was not only essential to decreasing the incidence of complications (e.g., hemorrhage, CSF leak), it was crucial to shortening the surgical duration, which correlated with lower surgery-related risks and higher expectations for early discharge. In our cohort, although CSF rhinorrhea occurred in one patient during surgery, it was not observed during the postoperative period or at the one month follow-up. Moreover, other potential severe complications such as pituitary dysfunction, visual dysfunction, meningitis, and major bleeding were not observed during the follow-up period. Our initial results may suggest a strong support of the safety and efficacy of treating nonfunctional pituitary adenoma with the EEA following an AS protocol.

In our protocol, effective and explicit education to patients and their caregivers was stressed to optimize recovery and reduce risks from preoperative counseling to postoperative follow-up. The protocol was implemented in an interactive way centered on patient preferences. Prior to enrollment, patients were expected to be discharged home on the operation day and were encouraged to achieve that by strictly following the enhanced recovery strategy. Preoperatively, patients were confirmed that their health and safety were prioritized by our surgical team. Meanwhile, significant aspects of the protocol were explained to the patients and their caregivers in an effort to boost the patient’s general status and lead to a shorter LOS, the routine aspects of the surgical procedure, and the potential risks and their management. Postoperatively, eligible patients and caregivers were reminded of the anticipation of early discharge and provided the knowledge required to recognize complications including DI, hyponatremia, CSF leak, visual decline, and other neurological dysfunction. Only patients with an acceptable general condition that met all of the discharge criteria were allowed to leave the hospital. In addition to the evaluations of neurological function and severe complications, the PADSS and KPS effectively promoted safety. In our cohort, 12 patients were discharged from hospital with a PADSS score of 9 and the other eight patients with a PADSS score of 10, while most of the patients’ KPS scores were 80, which indicated their ability to move and function normally. No patient was readmitted for severe complications. When patients were discharged, they were told to arrange follow-up at different period after surgery by telephone contact and face-to-face interview. In addition, a hotline and green-channel system were implemented to provide emergency medical treatment and psychological comfort whenever needed.

Adequate preoperative education is essential to promote earlier discharging. In our AS protocol, preoperative evaluation and education was performed by a specialized working group, in which clinicians from different department were included, for example, neurosurgeons, anesthetists, nutritionists, inpatient and operating room nurses. The eligibility of enrollment in AS protocol was assessed by a neurosurgeon and anesthesiologist together. Evaluations for nutritional and psychological status, and for the risk of PONV and other perioperative complications were also carefully performed. By communicating with the patients about the conditions in detail, possible prophylaxis measures were raised. Fully understanding the aim and procedures of AS operation in the following days, patients could perceive that discharging early on the operation day can be feasible and safe. This promotes their compliance and encourages them to prepare for discharging early both practically and psychologically.

For the perioperative management strategy, the main concept of the AS protocol followed in this study was inherited from the ERAS protocol for patients undergoing elective craniotomy that we developed previously^2,6,28–30^. After successful implementation of the ERAS protocol for craniotomy, we further revised and designed a specialized protocol for the EEA. By reviewing the current literature and integrating the AS trends, we finalized the AS protocol for endoscopic endonasal resection of pituitary adenomas which were presented in the prior part of this article. Although the general principles and basic concepts were similar to those of ERAS, the practical details followed the AS regime. Especially for patients with adenoma, we focused on several perioperative factors such as functional status evaluation and elevation, postoperative PONV risk assessment and prevention, surgical land anesthetic management, use of non-opioid analgesia, fluid balance, early fluid and food intake, and early ambulation^31–34^. In addition, to help achieve the target of same-day discharge, we made some modifications of the current postoperative workflow. In our study, patients who were assessed as having intact neurological status in the AS unit were not recommended for transfer to the intensive care unit after the operation but were encouraged to mobilize early and strive for early discharge.

One of the major safety concerns to both neurosurgeons and patients would be the prevention of DI and postoperative fluid and electrolytes disturbances. Functional adenomas with relatively large diameter, or with infiltrative growth pattern may related to a higher rate of complications. In our AS practice, a comparatively more strict including criteria was applied, in order to excluded those high-risk candidates in current regime. Though patients in our cohort were discharged with normal urine and serum assessment, DI can still be an influencing factor for emergency room visits and re-admission. Its incidence is usually transient and could be suspected with the daily follow-up communication with our AS workgroup specialists. In our cohort, no DI was reported, but screening for suspected manifestations of transient DI was important in daily follow-up. Especially on day 8, the assessment of urine and serum were required to further evaluate the fluid and electrolyte metabolism. It is worth noting that delayed hyponatremia is not uncommon in patients underwent EEA. Majority cases of this complication are monophasic and symptomatic, and reach their peak at 3–7 days after the operation. In this way, the combination of telephone follow-up and subsequent laboratory assessment by day 8 may be reasonable to detect the incidence of the complication^35^.

In this initial stage, reasonable or even conservative patient selection criteria and practical measurements in postoperative follow-up were also required. Compared with another pioneering work by Thomas *et al*., we followed a stricter preoperative evaluation to ensure suitable patient recruitment. The patients’ age, the tumor size, and the parasellar growth were all factors that associated with the incident of complications^27^. Also, tumor with Knosp grades 3–4 and a higher grade of intraoperative CSF leak could increase the risk of postoperative CSF leak^36^. Thus, in the current study, only nonfunctioning adenomas with a maximum diameter <3 cm, Knosp grade ≤2, and Hardy-Wilson grade ≤IIb were considered suitable for the AS.

Though the goal of discharging on the operation day was achieved in most patients in this cohort, we noticed that endocrine active adenoma and macroadenoma were excluded from this trial. The clinical prognosis benefit largely arise from the strict including and excluding criterion with current AS protocol. However, patients meeting this criterion account for relatively smaller part of whole patients with adenomas requiring surgical intervention. Hence our AS protocol still need further updates to extend its applicable range. Since there has been no randomized controlled trial (RCT) evaluating this AS protocol, the safety and efficacy of the current AS protocol need to be further investigated to reach a balance between short LOS and AS safety.

## Conclusion

Here we introduced an AS protocol for the resection of pituitary adenomas using an EEA to eligible low-risk patients. This initial trial showed that this evidence-based AS protocol for perioperative management could enable patients to be discharged on the operation day in most cases. The participants demonstrated a favorable prognosis during the follow-up period without severe early- or late-stage complications. Our results support the safety and efficacy of our AS protocol for pituitary adenoma patients undergoing EEA resection. However, further assessments of this protocol in large randomized controlled trials are warranted to confirm our findings.

## Supplementary information

Supplementary information.
