# Supplementary material for: Ambulatory Surgery Protocol for Endoscopic Endonasal Resection of Pituitary Adenomas: A Prospective Single-arm Trial with Initial Implementation Experience
Source: Sci Rep. 2020 Jun 16;10:9755. doi: 10.1038/s41598-020-66826-9 (PMC7297807; doi:10.1038/s41598-020-66826-9)
Supplement: Supplementary file 1 — Supplementary information. [file 41598_2020_66826_MOESM1_ESM.docx]

**Ambulatory Surgery Protocol for Endoscopic Endonasal Resection of Pituitary Adenomas: A Prospective Single-arm Trial with Initial Implementation Experience**

Yang Liu^2*^, MD, PhD. Tao Zheng^1,4*^, MD. Wenhai Lv^1^, MD. Long Chen^1^, MD. Binfang Zhao^1^, BSN. Xue Jiang^1^, BSN. Lin Ye^3^, MD. Liang Qu^1^, MD. Lanfu Zhao^1^, MD. Yufu Zhang^1^, MD. Yafei Xue^1^, MD. Lei Chen^1,4^, MD. Bolin Liu^1,4*^, MD, PhD. Yingxi Wu^1^, MD. Zhengmin Li^5^, MD. Jiangtao Niu^5^, MD. Ruigang Li^1,4^, BSN. Yan Qu^1^, MD, PhD. Guodong Gao^1^, MD, PhD. Yuan Wang^1#^, MD, PhD., Shiming He^1,4#^, MD, PhD.

1. Department of Neurosurgery, Tangdu Hospital, Fourth Military Medical University. Xi’an, China.

2. Department of Neurology, No.988 Hospital of Joint Logistic Support Force, Zheng Zhou, China.

3. Department of Nutrition, Tangdu Hospital, Fourth Military Medical University. Xi’an, China.

4. Department of Neurosurgery, Xi’an International Medical Center, Xi’an, China.

5. Department of Anesthesiology, Tangdu Hospital, Fourth Military Medical University. Xi’an, China.

* Yang Liu, MD, PhD, and Tao Zheng, MD contributed equally in this study.

#Corresponding Authors

Shiming He, MD., PhD.

Phone: +86 29 84717737

Fax:+86 29 84717737

Email:[he-shiming@163.com](mailto:he-shiming@163.com)

Yuan Wang, MD., PhD.

Phone: +86 29 84717838

Fax:+86 29 84717737

Email:[bladerunnerwang@qq.com](mailto:bladerunnerwang@qq.com)

**Key words**: Pituitary adenoma, ambulatory surgery, endonasal endoscopic resection

**Running Title**: Ambulatory protocol for pituitary adenoma with EEA

This study has been registered in the Chinese Clinical Trial Registry (http://www.chictr.org.cn/showproj.aspx?proj=20336) with registration number ChiCTR-ONC-17012019 (date of registration: 17/07/2017). This work was supported by China Natural Science Foundation (81572470 and 81601100).

**Supplementary material**

A total of 30 patients underwent EEA surgery in traditional non-AS protocol. Some of their demographic and hormonally active characters were introduced in Table 1. The mean maximum diameter of their adenomas was 3.3±0.7 cm with a range of 2.4-4.9 cm. Gross resection was achieved in 27 patients (90%) and the median surgical duration was 102 min (range, 75-141min). The median duration of postoperative hospital stay was 6.3d, with a range of 4.3 to 9.3 d.

On the early post-operative outcomes (1 month), several complications were observed, including hypopituitarism in 3 cases (10%), CSF leakage in 2 cases (7%), olfactory impairment in 4 cases (13%), diabetes insipidus in 1 case (3%), and electrolyte disturbance in 3 cases (10%). Most of them were confirmed within the first week after the operation. No intracranial infection case was observed during the hospital stay and follow-up period.

The adenoma relapsed in 1 patient who received non-AS endoscopy resection, which was confirmed 1 year after the operation by follow-up MRI examination. This was a prolactin secreting adenoma with a maximum diameter of 3.7 cm.

**Supplementary Table: Perioperative clinical features of 30 patients received EEA other than AS protocol**

| **Parameters** | **Values** |
| --- | --- |
| Mean maximum diameter (range, cm) | 3.3±0.7 (2.4-4.9) |
| Median duration of the operation in min (min, 1stQ, 3rd Q, max) | 102 (75,92,123,141) |
| Gross resection, no.( %) | 27 (90%) |
| Median duration of postoperative hospital stay (d, 1stQ, 3rd Q, max) | 6.3 (4.3, 5.8, 7.5, 9.3) |
| Follow-up postoperative complications (1 month) |  |
| Hypopituitarism | 3 (10%) |
| Cerebrospinal fluid leakage | 2 (7%) |
| Olfactory impairment | 4 (13%) |
| Diabetes insipidus | 1 (3%) |
| Electrolyte disturbance | 3 (10%) |
| Intracranial infection | 0 |
| Relapse (within 1 year) | 1 (3%) |

*EEA: endoscopic endonasal approach

AS: ambulatory surgery
